# Theta-frequency medial septal nucleus deep brain stimulation increases neurovascular activity in MK-801-treated mice

**DOI:** 10.3389/fnins.2024.1372315

**Published:** 2024-03-15

**Authors:** Lindsey M. Crown, Kofi A. Agyeman, Wooseong Choi, Nancy Zepeda, Ege Iseri, Pooyan Pahlavan, Steven J. Siegel, Charles Liu, Vasileios Christopoulos, Darrin J. Lee

**Affiliations:** ^1^Department of Psychiatry and Behavioral Sciences, Keck School of Medicine, University of Southern California, Los Angeles, CA, United States; ^2^Department of Bioengineering, University of California Riverside, Riverside, CA, United States; ^3^Department of Neurological Surgery, Keck School of Medicine, University of Southern California, Los Angeles, CA, United States; ^4^Neurorestoration Center, Keck School of Medicine, University of Southern California, Los Angeles, CA, United States; ^5^Viterbi School of Engineering, University of Southern California, Los Angeles, CA, United States; ^6^Rancho Los Amigos National Rehabilitation Center, Downey, CA, United States; ^7^Neuroscience Graduate Program, University of California Riverside, Riverside, CA, United States

**Keywords:** hippocampus, medial septal nucleus, deep brain stimulation, functional ultrasound imaging, MK-801, schizophrenia, theta

## Abstract

**Introduction:**

Deep brain stimulation (DBS) has shown remarkable success treating neurological and psychiatric disorders including Parkinson’s disease, essential tremor, dystonia, epilepsy, and obsessive-compulsive disorder. DBS is now being explored to improve cognitive and functional outcomes in other psychiatric conditions, such as those characterized by reduced N-methyl-D-aspartate (NMDA) function (i.e., schizophrenia). While DBS for movement disorders generally involves high-frequency (>100 Hz) stimulation, there is evidence that low-frequency stimulation may have beneficial and persisting effects when applied to cognitive brain networks.

**Methods:**

In this study, we utilize a novel technology, functional ultrasound imaging (fUSI), to characterize the cerebrovascular impact of medial septal nucleus (MSN) DBS under conditions of NMDA antagonism (pharmacologically using Dizocilpine [MK-801]) in anesthetized male mice.

**Results:**

Imaging from a sagittal plane across a variety of brain regions within and outside of the septohippocampal circuit, we find that MSN theta-frequency (7.7 Hz) DBS increases hippocampal cerebral blood volume (CBV) during and after stimulation. This effect was not present using standard high-frequency stimulation parameters [i.e., gamma (100 Hz)].

**Discussion:**

These results indicate the MSN DBS increases circuit-specific hippocampal neurovascular activity in a frequency-dependent manner and does so in a way that continues beyond the period of electrical stimulation.

## Introduction

Deep brain stimulation (DBS) is a form of neuromodulation that has shown remarkable success reducing symptomology and improving functional outcomes in neurological conditions such as movement disorders, epilepsy, and even Alzheimer’s disease (Laxton et al., 2010; Dougherty et al., 2016). There is now a growing interest in utilizing DBS to treat cognitive impairment associated with neurological and psychiatric disorders. While currently an area of active research, how DBS alters brain activity in networks associated with cognitive function is yet to be fully elucidated (Lozano et al., 2019).

Functional ultrasound imaging (fUSI) is a relatively new technology that enables large-scale estimates of neural activity through measures of cerebral blood volume (CBV) changes. Specifically, fUSI measures the power Doppler (pD) signal across a wide spatial field (~1 cm in-plane and up to 5 cm in-depth), making it well-suited for wide-scale imaging of the rodent brain. fUSI measures changes in cerebral blood volume by detecting backscattered echoes from red blood cells moving within its field of view. Recent studies aimed to establish the direct association between neuronal activity and pD signal provide evidence that low-frequency fluctuations (below 0.3 Hz) in pD signal are strongly correlated with neuronal activity in rodents (Nunez-Elizalde et al., 2022). Importantly, fUSI provides a unique combination of high spatiotemporal resolution (~100 μm, ~ 100 ms) and high sensitivity to slow blood flow (~1 mm/s velocity). fUSI has already been proven to be an effective tool for imaging large-scale brain activity and pharmacodynamics (Macé et al., 2011, 2013; Rabut et al., 2020; Norman et al., 2021). As such, it is well-positioned to improve our understanding of the impact of DBS on CBV in brain networks during and immediately following stimulation.

The medial septal nucleus (MSN) is a key structure in the septohippocampal network that modulates sensory-motor processing and acts as a “pacemaker” for hippocampal theta oscillations via dense glutamatergic, cholinergic, and GABAergic projections to the hippocampus (Fuhrmann et al., 2015; Leão et al., 2015). This relationship with the hippocampus makes the MSN a promising target for DBS in cognitive disorders involving memory impairments (Takeuchi et al., 2021). Our recent work as well as that of others suggest that modulating the septohippocampal network via MSN DBS can restore cognitive impairment and memory dysfunction in preclinical models of epilepsy, traumatic brain injury, Alzheimer’s disease, and schizophrenia (Lee et al., 2013, 2015, 2017; Takeuchi et al., 2021; Cole et al., 2022; Zepeda et al., 2022). Indeed, in a recent paper by our group, we found that MSN theta (7.7 Hz) but not gamma (100 Hz) stimulation was able to rescue memory impairments in animals treated with MK-801, a potent and selective NMDA receptor antagonist often used to model schizophrenia (Zepeda et al., 2022). Specifically, we observed that theta stimulation improved Barnes maze performance in MK-801-treated animals, but not saline-treated animals. Furthermore, gamma-stimulated saline-treated animals performed significantly worse than non-stimulated animals. These divergent effects of stimulation frequency on memory performance led to us to question how low and high frequency stimulation of the MSN might differ in their impact on brain networks.

**Figure 1:**
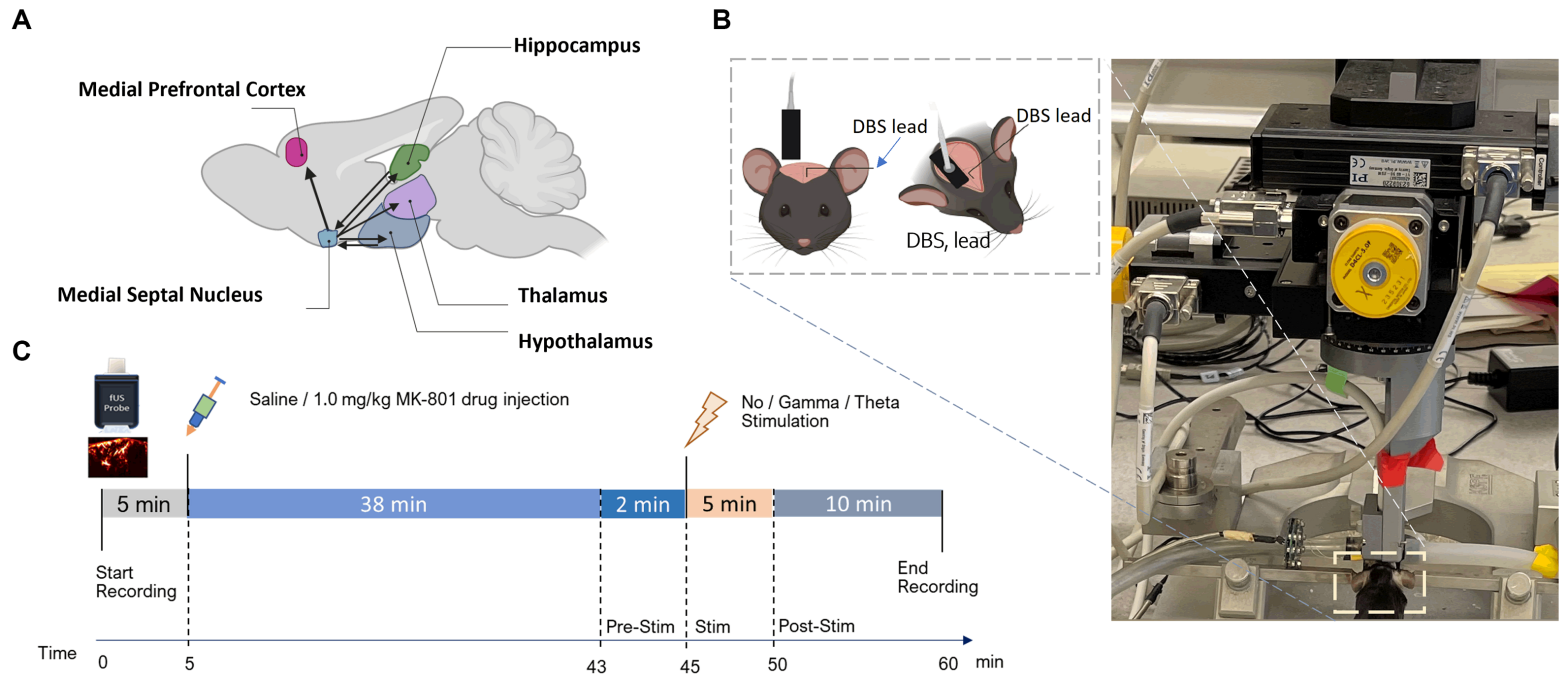
Experimental setup and fUSI recording protocol. **(A)** Schematic illustration of connectivity between the MSN and ROIs. Arrowheads represent axonal projections to and/or from MSN. **(B)** Experimental set-up showing the anesthetized mouse in a stereotaxic frame under the Iconeus One motorized probe mount. DBS stimulating electrodes were implanted on the left hemisphere and a sagittal plane of the right hemisphere was imaged. **(C)** Diagram of the protocol for the 60 min of continuous fUSI acquisition. After 5 min, saline or 1.0 mg/kg MK-801 was injected. After a total of 45 min of either theta, gamma, or no stimulation was applied for 5 min followed by 10 more minutes of recording.

In the current study, we utilize fUSI to characterize the effects of MK-801 on CBV changes (ΔCBV) in the anesthetized mouse within the septohippocampal network including the hippocampus and medial prefrontal cortex (mPFC) (Figure 1A). Within the same sagittal plane, we also compute ΔCBV in regions of interest (ROIs) outside this network including the striatum, thalamus, hypothalamus, and pallidum. Note that other regions connected with the MSN, such as amygdala, habenula, or raphe nucleus were not recorded, since they were not accessible from the selected 2D imaging plane. Finally, we test the hypothesis that MSN theta-frequency stimulation can increase CBV under conditions of reduced NMDA function.

## Materials and methods

### Animals and surgical procedures

Eighty two male 8–12-week-old C57BL/6 mice (Charles River Laboratories; Hollister, CA) were used in this study. fUSI data from 2 animals were excluded due to extreme values (Grubbs test for outliers, 98th percentile of all maximum change in pD intensities) that did not appear physiological due to high amplitude fluctuations in pD (Grubbs, 1969; Stefansky, 1972). The animals were divided into 2 main groups: saline vehicle control (*n* = 44) and MK-801 drug-administered (*n* = 36). Each group was then sub-divided into 3 categories: no stimulation (saline: *n* = 14; MK-801: *n* = 10), theta stimulation (saline: *n* = 16, MK-801: *n* = 12), and gamma stimulation (saline: *n* = 14, MK-801: *n* = 14).

Mice were anaesthetized with 5% isoflurane in O_2_/N_2_O (1:2) carrier gas and then maintained at a constant rate (1.5–2%) through surgery and data acquisition. Body temperature was regulated throughout recordings by placing animals on an electric heating pad. Hair was removed from the mouse’s head using a commercially available depilatory cream (Nair, Pharmapacks).

To implant DBS electrodes, mice were head-fixed in a stereotaxic frame (David Kopf instruments, Tujunga, CA) and a midline incision of the scalp was made to expose the skull. A 2 mm burr hole was then drilled to implant a twisted-pair bipolar stimulating electrode (E363T/2/SPC ELEC 0.008″/.2MM, Plastics One Inc., Roanoke, VA) targeting the midline MSN (AP: +0.7 mm, ML: −0.9 mm, from bregma. Z: −4.39 mm at 11.8 degrees) from the left hemisphere. Prior to implantation, the electrodes were connected to an electronic interface board (Neuralynx Inc., Bozeman, MT) and bent at 4.5 mm from the tip to maximize the proximity of the fUSI probe to the skull (Figure 1B, inset). All procedures were approved by the University of Southern California, Institutional Animal Care and Use Committee (IACUC #21006).

#### Histology for electrode placement confirmation

**Figure 2:**
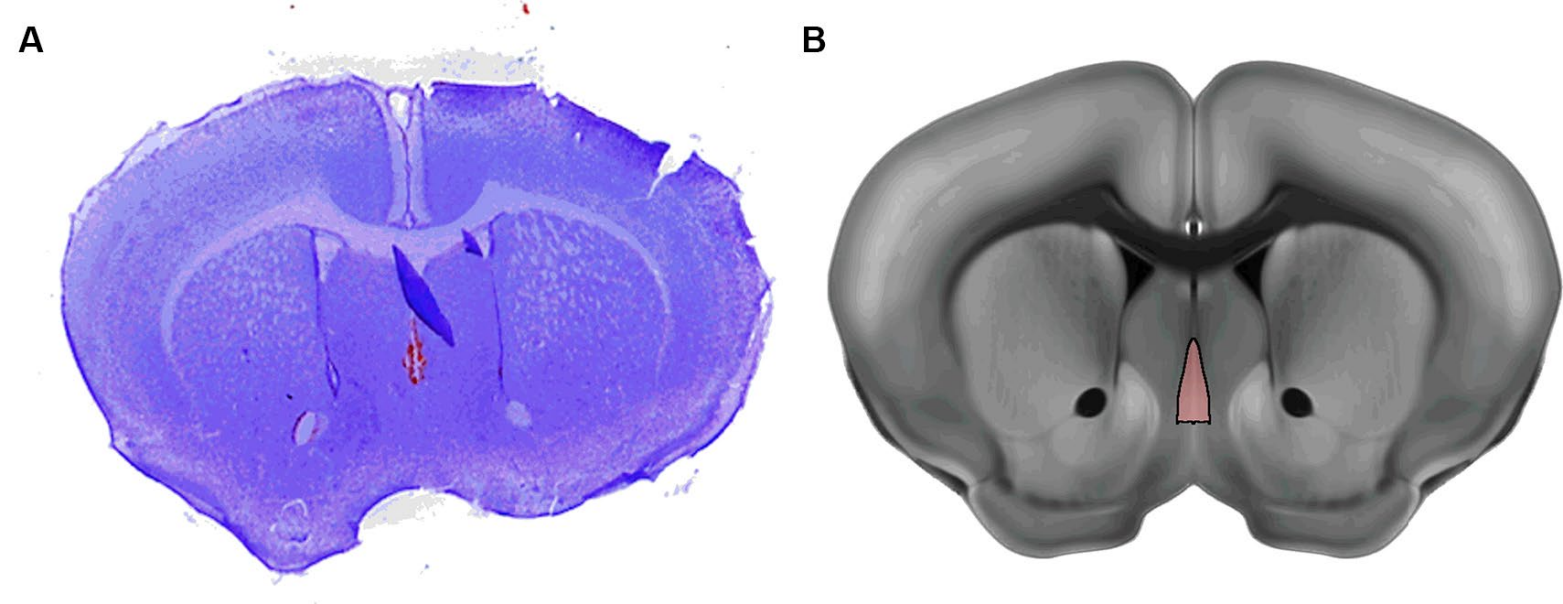
Example of histological mapping of electrode placement in the MSN. **(A)** Representative Nissl stain of the mouse brain with lesion indicating the electrode placement **(B)** Annotation of the MSN from the Allen Reference Atlas shows the MSN in approximately the same position as the lesion in **A**.

Following the recording, the brain was lesioned at the electrode tip by applying 1 mA of current for 5 s. Mice were then transcardially perfused with 50 mL of 0.1 M phosphate buffered saline (PBS) and 50 mL of 4% paraformaldehyde. Brains were harvested and stored in PBS at 4°C. 100 μm Coronal sections were cut with a vibratome (Leica VT 1200; Leica Biosystems, Buffalo Grove, IL) and then Nissl stained with Cresyl Violet and imaged with a high resolution scanner to confirm electrode positioning and exclude data from incorrectly placed electrodes (Figures 2A,B).

#### Data acquisition

fUSI data were acquired using the *Iconeus One* system (Iconeus, Paris, France). Image acquisition was performed using a 128-channel linear ultrasound transducer array, with 15 MHz center frequency and 0.1 mm pitch. This technique enables a large field of view (12.8 mm width, 10 mm depth and 400 μm plane thickness) with a spatial resolution of 100 μm x 100 μm in-plane. The transducer was placed on the intact skull and skin along a sagittal plane on the right side, contralateral to the implanted stimulating electrodes (Figure 1B). fUSI was continuously recorded for 60 min in the sagittal plane to image the septohippocampal network (Figure 1C). After the first 5 min of recording, an intraperitoneal (i.p) injection of either 0.2 cc of saline or MK-801 (1.0 mg/kg) was given using a butterfly needle. To reduce the chance of the injection creating motion artifacts, the butterfly needle was inserted prior to recording. 1.0 mg/kg of MK-801 has been used in previous studies and found to produce schizophrenia- like deficits in mice (Long et al., 2006; Olszewski et al., 2008; Xu et al., 2022). At the 45-min mark, mice received either 5 min theta (7.7 Hz), gamma (100 Hz), or no stimulation in the MSN, followed by an additional 10 min of fUSI recording (Figure 1C). This timepoint was chosen as the maximal concentration of MK-801 in the brain when given systemically occurs 40–60 min after injection (Wegener et al., 2011). The MSN was electrically stimulated at gamma and theta frequency with a 100 msec square wave pulse width and current of 80 μA using a STG 4008 (Multi-Channel Systems, Baden-Württemberg, Germany). These parameters were chosen because because they have previously been investigated in awake behaving animals (Lee et al., 2013, 2017; Zepeda et al., 2022).

**Figure 3:**
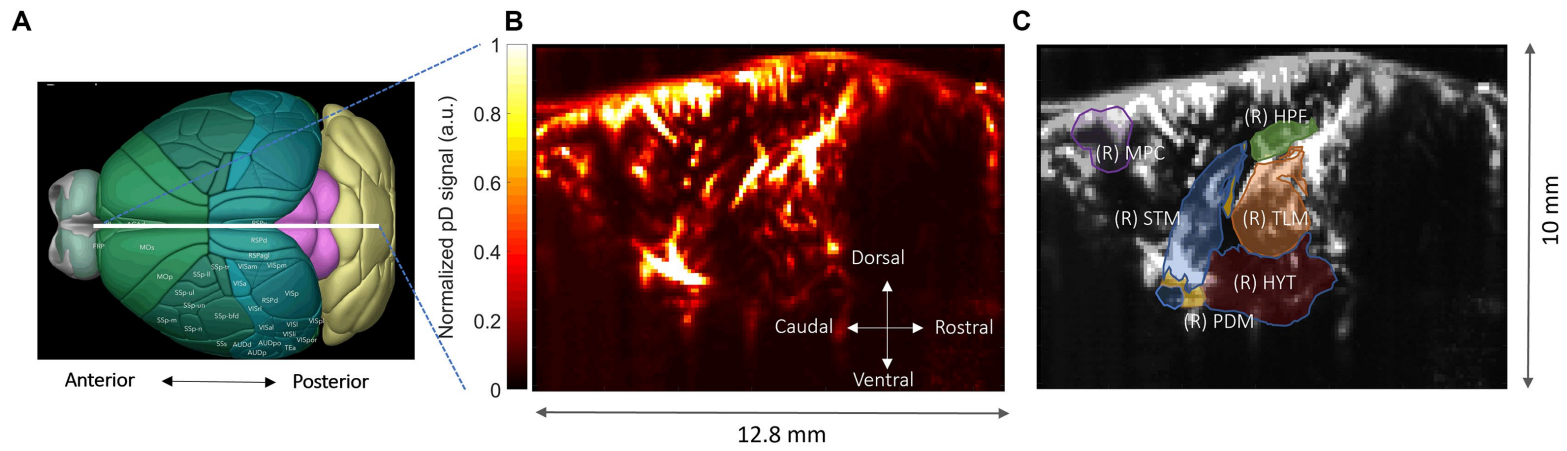
Functional ultrasound imaging of the mouse brain. **(A)** 3D mouse brain model with fUSI probe positioning (white bar). **(B)** Power Doppler-based vascular maps in a sagittal plane (max-min normalized relative scale). **(C)** ROIs – hippocampus (HPF), medial prefrontal cortex (mPFC), hypothalamus (HYT), thalamus (TLM), pallidum (PDM), and striatum (STM), superimposed onto a mean grayscale fUSI vascular map of the sagittal mouse brain.

The target image plane was determined by co-registering a 3D whole-brain fUSI image of each mouse with a standard Allen Mouse Common Coordinates Framework brain atlas utilizing dedicated software available with the Iconeus One system (Figure 3A; Wang et al., 2020). The probe was fixed steadily throughout experiments on a motorized system with the field of view (FOV) transverse and intersecting the co-registered sagittal plane. Images were obtained from 200 compounded frames acquired with a 500 Hz frame rate, using 11 tilted plane waves separated by 2 degrees (from −10° to +10° incremented by 2°). These were acquired at a 5.5 kHz pulse repetition frequency (PRF) using a real-time continuous acquisition of successive blocks of 400 ms (with 600 ms pause between pulses) of compounded plane wave images.

### Data analysis

#### Data pre-processing

The Iconeus One acquisition system generates pD images pre-processed with built-in phase-correlation based sub-pixel motion registration and singular-value-decomposition (SVD) based clutter filtering algorithms (Ledoux et al., 1997). These algorithms were used to separate tissue signal from blood signal to obtain pD images (Figure 3B). To correct potential physiological and motion artifacts, we adopted rigid motion correction techniques that have successfully been used in fUSI and other neuroimaging studies (Pnevmatikakis and Giovannucci, 2017; Stringer and Pachitariu, 2019; Friedrich et al., 2021). These were combined with high frequency filtering algorithms to eliminate noise artifacts. We employed a low-pass filter with normalized passband frequency of 0.02 Hz, with a stopband attenuation of 60 dB that compensates for delay introduced by the filter to remove high-frequency fluctuations in the pD signals.

#### Effects of MK-801 and DBS on CBV

All analyses were performed in Matlab Version 9.13.0.2193358 (R2022b). To investigate the temporal effects of intraperitoneal MK-801 administration on the septohippocampal circuit and surrounding regions, we computed ΔCBV as a percentage change of the pD signal from baseline activity for the selected ROIs. The average pD signal from 2 min prior to saline or drug injection was used as the baseline. We utilized a repeated measures analysis of variance (rmANOVA) to assess the effects and interactions between drug (saline and MK801) and ROI over time. We fitted a repeated measures ‘*within-design’* model to the CBV percentage change signals over a 42-min interval (including 2 min just before the drug injection and the 40 min after injection) for each mouse and ROI. To further quantify the relative differences in ΔCBV between saline-vehicle and MK-801-treated mice in ROIs, we used the last 2 min of recordings to compute the mean effects-size differences in ΔCBVs and the 95% confidence interval of the effect size (if 95% confidence interval contains zero, then the effect is not significant at the *p* < 0.05) in each ROI. We also computed the Cohen’s *d* value in each ROI as a measure of the drug effect size that describes the standardized difference between the means of ΔCBVs in the two groups of animals (Liu, 2013). A Cohen’s d value of 0.2 represents a small effect size, 0.5 represents a moderate effect size, 0.8 represents a large size and greater than 0.8 represents a very large size (Lakens, 2013).

To determine the effects of stimulation on hemodynamics we utilized a three-way rmANOVA to assess the effects and interactions between drug (saline and MK801), stimulation (gamma, theta, no-stimulation) and ROI across time during the stimulation process. The mean ΔCBVs were calculated utilizing the last 2 min of pD signal during stimulation across animals in each of the three stimulation categories. Similarly, to determine the effects of MSN DBS after stimulation, we repeated the same analysis described above, but used the 10 min of pD signal after stimulation offset and 2 min prior to stimulation onset as a baseline.

## Results

### N-methyl-D-aspartate antagonist MK-801 reduced CBV relative to saline control

**Figure 4:**
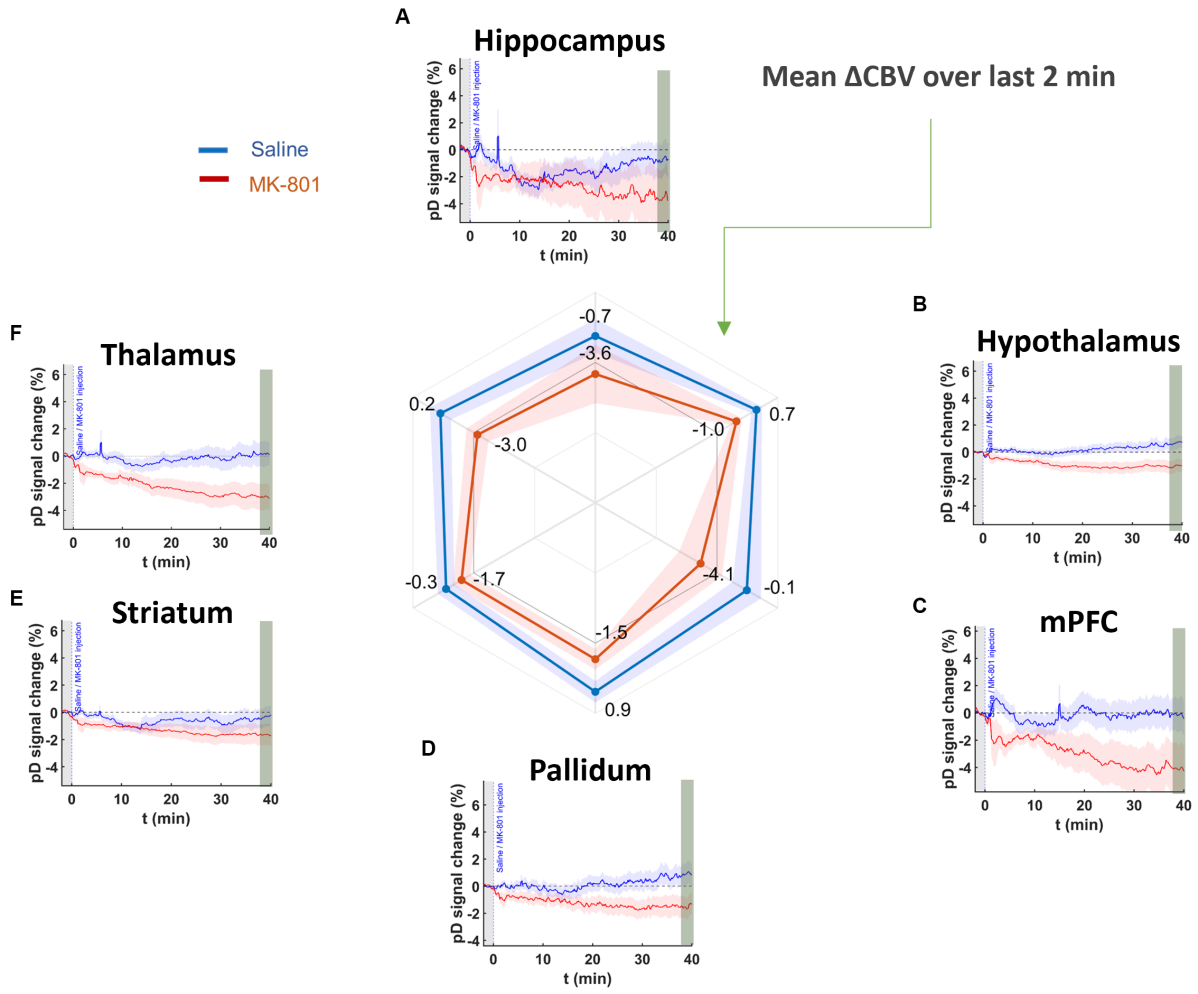
pD signal change (i.e., ΔCBV) in ROIs prior to stimulation onset in saline- and MK-801-treated animals. Curves show for **(A)** hippocampus, **(B)** hypothalamus, **(C)** mPFC, **(D)** pallidum, **(E)** striatum, and **(F)** thalamus after saline (blue) and 1.0 mg/kg MK-801 (red) injection. The radar chart shows that MK-801-induced decreases in CBV in all ROIs more than saline over the last 2 min (38–40 min post injection).

We analyzed 40 min of pD signal from the septohippocampal circuit (hippocampus, mPFC) as well as surrounding structures (hypothalamus, thalamus, pallidum, and striatum) to assess the effects of MK-801 on brain hemodynamics (Figure 4). A two-way repeated measures ANOVA (treatment × ROI) revealed that there was a significant main effect of drug [*F* (2,519, 56,406) = 8.76, *p* = 6.3 ×10^−5^] after Greenhouse-Geiser approximation correction. The effects of saline and MK-801 on the pD signal change (i.e., ΔCBV) (mean ± SEM) for each RO1 are given in Table 1 and reflect the average of the last 2 min before stimulation minus the baseline. The time courses of the pD signal change can be seen in Figure 4. We found that MK-801 induces greater decreases in CBV than saline control in all ROIs investigated. Specifically, by region these were: mPFC (3.96 ± 0.38%, *d* = 0.42), thalamus (3.17 ± 0.23%, *d* = 0.55), hippocampus (2.82 ± 0.42%, *d* = 0.27), pallidum (2.42 ± 0.20%, *d* = 0.49), hypothalamus (1.72 ± 0.12%, *d* = 0.59) and striatum (1.33 ± 0.18%, *d* = 0.29). Together, these results indicate that MK-801 reduces CBV both within and outside of the septohippocampal network.

**Table 1:** The effects of Saline and MK-801 on ΔCBV.

Region	Saline (% change)	MK-801 (% change)
Hippocampus	−0.7 ± 0.6	−3.6 ± 1.1
mPFC	−0.1 ± 0.2	−4.1 ± 0.61
Hypothalamus	−0.7 ± 0.6	−1.0 ± 0.2
Thalamus	0.2 ± 0.4	−3.0 ± 0.5
Striatum	−0.3 ± 0.4	−1.7 ± 0.3
Pallidum	0.9 ± 0.4	−1.5 ± 0.4

Values reflect the mean ΔCBV of the last 2 min before stimulation minus the mean ΔCBV of the baseline period +/− SEM.

### Effects of MSN stimulation on CBV in saline control and MK-801-treated animals

**Figure 5:**
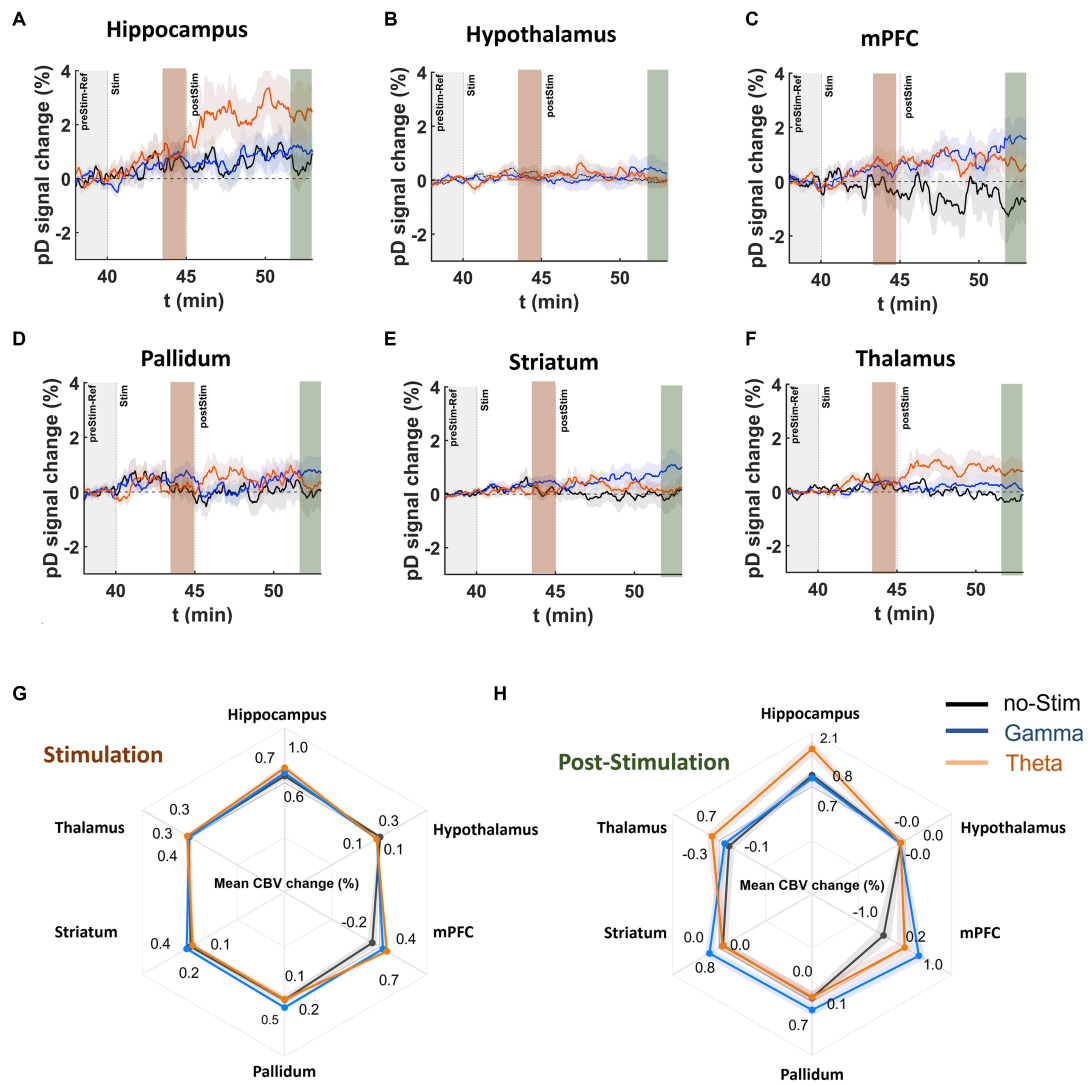
DBS effects on pD signal during and post stimulation onset in saline-treated animals. **(A–F)** Temporal course (theta [orange], gamma [blue], no-stimulation [black]) of mean pD signal change (i.e., ΔCBV) relative to baseline for the **(A)** hippocampus, **(B)** hypothalamus, **(C)** mPFC, **(D)** pallidum, **(E)** striatum, and **(F)** thalamus regions in the saline-treated animals. **(G,H)** Radar charts illustrate the mean %pD change during and post stimulation for theta-, gamma-, and no-stimulation animals in the ROIs investigated. Means were calculated using the last 2 min of pD signals acquired during (3rd – 5th minute after stimulation offset) and post (8th – 10th minute after stimulation offset) stimulation, respectively, across animals in each stimulation category.

First, we assessed whether theta- and gamma-frequency MSN stimulation have different impacts on CBV measures in saline-treated control and MK-801-treated mice. pD signal changes reflect the temporal responses of ΔCBV to theta, gamma, and no stimulation during and stimulation (Figures 5A–F). A three-way repeated measures ANOVA found a significant effect of drug (*F*(419, 186,036) = 7.35, *p* = 1.85 × 10^−8^), and stimulation (*F*(823, 186,036) = 2.71, *p* = 7.20 ×10^−4^) over time, as well as an interaction of drug and stimulation over time (*F*(838, 186,036) = 1.96, *p* = 1.95 ×10^−2^) during the 5 min stimulation interval, after Greenhouse–Geisser approximation correction.

Comparing stimulation frequency, we found that MSN theta stimulation increased CBV compared to no stimulation only in the mPFC (mean ΔCBV difference between theta- and no-stimulation ± confidence, Cohen’s d; 0.82 ± 0.12%, d = 0.45) and hippocampus (0.39 ± 0.12%, d = 0.21). For the rest of the ROIs the effect size magnitude was either very small (i.e., Cohen’s d < 0.08) or theta-frequency stimulation caused further reduction in CBVs compared to no-stimulation.

MSN gamma stimulation increased CBV compared to no-stimulation in the mPFC (0.60 ± 0.11%, *d* = 0.36), pallidum (0.38 ± 0.09%, d = 0.30) and striatum (0.22 ± 0.06%, *d* = 0.26; Figure 5G). For the rest of the ROIs, the effect size magnitude was either very small (i.e., Cohen’s d < 0.095) or gamma-frequency stimulation resulted in further reduction in CBVs compared to no-stimulation. Comparing the ΔCBV induced by the theta and gamma stimulation in mPFC – the only ROI that exhibited moderate effects for both types of stimulations – we found a very small effect size for ΔCBV between the two types of stimulation (mean ΔCBV differences between theta- and gamma-stimulation ± confidence, Cohen’s d; 0.22 ± 0.10, d = 0.14).

### Effects of MSN stimulation on CBV in the post-stimulation period

To determine the effects of MSN DBS after stimulation offset, we conducted a three-way repeated measures ANOVA (treatment × ROI × DBS) over the 10 min period after stimulation. We found significant effects of drug over time [*F* (1,019, 452,436) = 5.28, *p* = 1.27 ×10^−4^], stimulation over time [*F* (2038, 452,436) = 3.67, *p* = 1.17 ×10^−4^], as well as an interaction of drug and stimulation over time [*F* (2038, 186,036) = 3.09, *p* = 9.40 ×10^−4^]. To determine the effect size, we computed the mean ΔCBV in the last 2 min of the acquisition (8-10 min post-stimulation) and compared the mean-effect size differences on ΔCBV between theta, gamma or and no stimulation in each ROI. The results showed that MSN theta stimulation significantly increased CBV compared to no-stimulation in the hippocampus (1.30 ± 0.21, d = 0.42), mPFC (1.20 ± 0.22%, *d* = 0.37) and thalamus (0.97 ± 0.11%, *d* = 0.58; Figure 5H – radar chart).

MSN gamma stimulation resulted in a CBV increase compared to no stimulation in the mPFC (2.01 ± 0.22%, *d* = 0.60), striatum (0.77 ± 0.14%, *d* = 0.37) and pallidum (0.61 ± 0.16%, *d* = 0.25). Comparing the differences in ΔCBV induced by theta and gamma stimulation in the mPFC, the only ROI that exhibited a medium effect for both stimulation frequencies, we found that gamma induces a higher ΔCBV than theta stimulation with a medium effect size difference (0.81 ± 0.17, *d* = 0.31).

### Effects of stimulation frequency on CBV in MK-801-treated animals

**Figure 6:**
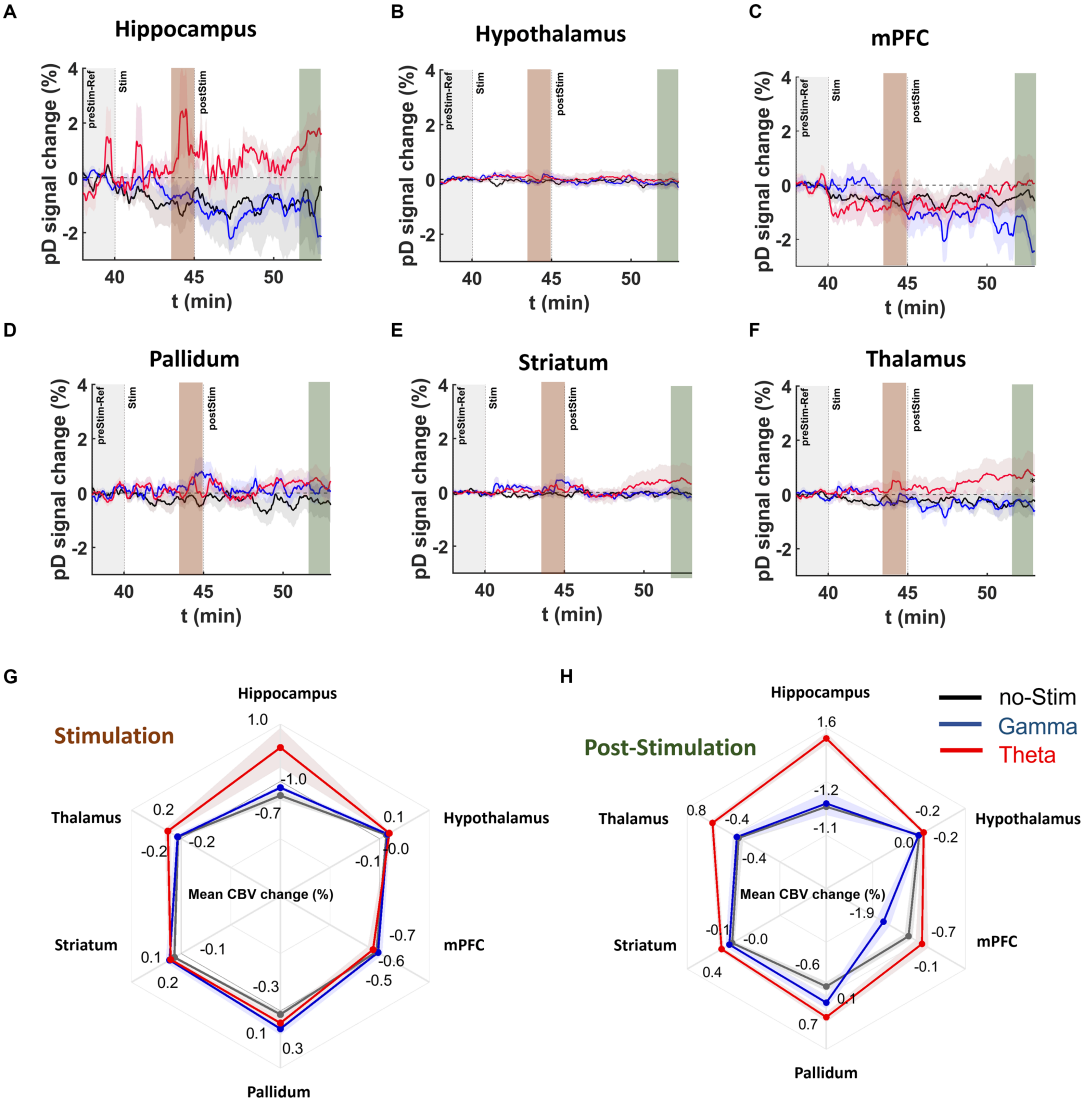
DBS effects on pD signal during and post stimulation onset in MK-801 treated mice. **(A–F)** Temporal course [theta (red), gamma (blue), no-stimulation (black)] of mean pD signal change (i.e., ΔCBV) relative to baseline for **(A)** hippocampus, **(B)** hypothalamus, **(C)** mPFC, **(D)** pallidum, **(E)** striatum, and **(F)** thalamus regions in the MK-801 drug injected mice. **(G,H)** Radar charts present the mean percentage ΔCBVs during and after stimulation for theta [red], gamma [blue], and no-stimulation [dark gray] animals in the ROIs investigated. Means were calculated utilizing the last 2 min of pD signal during and post stimulation.

Recently, our group demonstrated that theta, but not gamma frequency DBS of the MSN improves spatial memory in MK-801 treated rats (Zepeda et al., 2022). Therefore, we sought to determine if MSN theta- and gamma-frequency stimulation had differing impacts on neurovascular activity measures within memory-associated regions including the mPFC and hippocampus and neighboring regions outside the septohippocampal network (striatum, pallidum, thalamus, hypothalamus) following MK-801 administration. Figure 6 displays ΔCBV for ROIs during stimulation in MK-801 treated animals. We found that MSN theta stimulation in the MK-801 treated group caused increased CBV relative to the no-stimulation group with a medium to large effect size in all ROIs except the except the mPFC; including the hippocampus (2.01 ± 0.20%, *d* = 0.78), thalamus (0.49 ± 0.07%, *d* = 0.54), pallidum (0.36 ± 0.07%, *d* = 0.43), striatum (0.18 ± 0.04%, *d* = 0.31) and hypothalamus (0.14 ± 0.04%, *d* = 0.30; Figure 6G). Comparing gamma stimulation to no stimulation during the stimulation period, we found only a medium effect-size increase in ΔCBV for the pallidum (0.60 ± 0.08%, *d* = 0.53) and striatum (0.23 ± 0.05%, *d* = 0.38). Effect sizes were small in all other ROIs.

Importantly, we found that after stimulation offset, effect sizes for ΔCBV in theta- compared to no-stimulation animals were medium in the pallidum (1.26 ± 0.15%, *d* = 0.66), hippocampus (2.8 ± 0.37%, d = 0.60) and thalamus (1.24 ± 0.17%, *d* = 0.57) and small to medium in the striatum (0.50 ± 0.13%, *d* = 0.31), hypothalamus (0.21 ± 0.06%, *d* = 0.28) and mPFC (0.64 ± 0.22%, *d* = 0.22; Figure 6H). For gamma stimulation compared to no stimulation, we found only small effects in the pallidum (0.66 ± 0.13%, *d* = 0.39; Figure 6H). For the remainder of the ROIs, the effect size was either very small (i.e., Cohen’s d < 0.1) or gamma-frequency stimulation resulted in further CBV reduction (i.e., in hypothalamus and mPFC) compared to no stimulation.

Additionally, our results showed medium or large mean effect size differences in ΔCBV between the theta- and gamma-stimulated MK801 groups after stimulation offset in the hippocampus (2.67 ± 0.28%, *d* = 0.70), mPFC (1.83 ± 0.23%, *d* = 0.59), and thalamus (1.15 ± 0.14%, *d* = 0.56; Figure 6H). Together these results demonstrate that theta-frequency stimulation elicits the strongest CBV response in MK-801-treated mice in the hippocampus and pallidum, while gamma stimulation had almost no effect on hippocampal CBV (Cohen’s d = 0.03) and decreased CBV in the mPFC compared to no-stimulation mice.

## Discussion

The present study utilized the high spatiotemporal resolution and sensitivity of fUSI to demonstrate that acute administration of MK-801 causes a significant reduction in CBV across all ROIs. Furthermore, we demonstrated that theta-frequency MSN DBS has a significant effect on the septohippocampal network, with the strongest effect on the hippocampus. Intriguingly, the observed increase in hippocampal CBV remained even after cessation of DBS. On the other hand, structures outside the septohippocampal network, such as the hypothalamus and striatum, show less of a response to theta-frequency DBS. These effects were less pronounced with gamma-frequency stimulation. These findings suggest that MSN theta-frequency DBS can provide relatively specific neuromodulation to the septohippocampal network.

### MK-801-801 reduced CBV in all ROIs

MK-801 and other NMDA antagonists have been widely used in preclinical models to mimic the behavioral and electrophysiological deficits associated with schizophrenia (Newcomer et al., 1999; Farber, 2003; Saunders et al., 2012; Balu, 2016). However, how MK-801 alters regional CBV in such models is not well known. We observed that MK-801 reduced CBV across all ROIs (Figure 4). Importantly, previous fMRI studies have observed reduced BOLD signals in hippocampal and prefrontal areas in schizophrenia patients (MacDonald et al., 2006; Gur and Gur, 2010; Bedford et al., 2012). In this context, our findings support the use of MK-801 as a neurovascular model of schizophrenia. Furthermore, our study demonstrates the feasibility of using fUSI to identify network-specific hemodynamic changes as an additional modality for studying neurocognitive disorders.

### Medial septal nucleus theta and gamma stimulation showed different regional response profiles

We observed that for both saline- and MK-801- treated animals, theta-frequency MSN DBS resulted in an increase to CBV in some ROIs but not others. Importantly, this effect was greatest in the hippocampus, which receives dense, direct projections from the MSN, and is a primary target for neuromodulatory interventions to treat cognitive dysfunction (Lee et al., 2013, 2015, 2017; Zepeda et al., 2022). Increased CBV during and after stimulation was also observed in the mPFC and thalamus, though only in the post-stimulation period (Figure 5). One possible reason increased CBV was observed in the thalamus only after stimulation is that this was an indirect effect of increased neurovascular activity in the hippocampus and mPFC. The thalamus receives dense, often recurrent projections from the hippocampus and mPFC (Aggleton et al., 2010; Bonjean et al., 2011). It is possible that a buildup of activity in these two regions was required to trigger a delayed, more gradual increase in thalamic CBV. Contrastingly, gamma stimulation did not alter hippocampal CBV nor did it strongly affect any other regions. Only the mPFC and striatum showed medium or large effects in response to gamma stimulation.

DBS for movement disorders, Parkinson’s disease in particular, has been utilized clinically for over 35  years and its mechanism of action in this context has been well-studied (Montgomery and Gale, 2008; Agnesi et al., 2013; Jakobs et al., 2019). In this application, DBS is usually administered at higher frequencies (~100-130 Hz) and has often been considered a ‘functional lesion’ (Benabid et al., 1988; Tóth and Tomka, 2007). It is now known that this effect is likely moderated by the short inter-pulse interval inherent with such frequencies preventing neurons from returning to their baseline activity (Jakobs et al., 2019). Contrastingly, low frequency (<60 Hz) DBS has been shown to entrain neurons (McConnell et al., 2012). Because this tends to exacerbate symptoms in movement disorders, low frequency DBS is not commonly used. However, low frequency oscillations, theta in particular (5–12 Hz), are crucial for cognitive processes and deficits in theta are linked to many neuropsychiatric conditions (Kane et al., 2019; Adams et al., 2020; Wirt et al., 2021; Knight et al., 2022). Furthermore, lesioning the MSN ablates hippocampal theta rhythmicity and restoring it improves memory (Petsche et al., 1962; McNaughton et al., 2006). Thus, one possibility is that MSN theta stimulation may increase hippocampal CBV by entraining neurons and thereby augmenting neurovascular activity. Correspondingly, the effects of gamma stimulation may also depend on the intrinsic firing properties of the region. For example, the striatum is home to a population of fast-spiking interneurons that have been shown to entrain to gamma (Higgs and Wilson, 2019). This may have played a role in the observed CBV increase for gamma- but not theta-frequency DBS in the striatum (Figure 5).

### Medial septal nucleus theta stimulation increased hippocampal CBV during and after stimulation despite NMDA antagonism

A leading hypothesis is that reduced N-methyl-D-aspartate (NMDA) receptor-mediated glutamatergic transmission underlies psychiatric conditions such as schizophrenia, which is often accompanied by cognitive and memory dysfunction (Newcomer et al., 1999; Farber, 2003; Moghaddam and Javitt, 2012; Saunders et al., 2012; Lin et al., 2014; Balu, 2016). As such, pharmacologic NMDA receptor antagonism (i.e., via MK-801 or ketamine) has been used in many studies to model schizophrenia and results in characteristic changes to neural oscillatory patterns and memory dysfunction (Spangler et al., 1991; Newcomer et al., 1999; Korotkova et al., 2010; Saunders et al., 2012; Billingslea et al., 2014).

We observed that MK-801 reduced CBV across all ROIs, however the greatest magnitudes were seen in the hippocampus and mPFC (Figure 5). Our previous work suggests that MSN theta, but not gamma stimulation can improve spatial memory in MK-801-treated rodents (Zepeda et al., 2022). While MSN theta stimulation increased hippocampal CBV during and after stimulation in MK-801-treated animals, this was not true of MSN gamma stimulation. Gamma stimulation resulted in delayed increases to mPFC CBV in saline-treated animals and had no effect on MK-801-treated animals in any of the ROIs. These results suggest that MSN gamma stimulation is not sufficient to engage hippocampal activity and highlights the importance of frequency parameters in DBS paradigms for neurocognitive disorders.

### Medial septal nucleus DBS resulted in increased CBV after the cessation of stimulation

One intriguing result from the present study is that regions showing an increase in CBV following stimulation, such as the hippocampus and thalamus, continued to show increased CBV after the stimulation was turned off (Figure 6). While in some instances DBS has been known to improve motor function beyond the period of stimulation, the success of DBS in movement disorders is usually evaluated with respect to its acute effects (Wolf et al., 2021). However, in studies of DBS for psychiatric conditions, the relationship between acute stimulation and symptom abatement is less clearly defined and may involve slower or more gradual changes to brain function (Abelson et al., 2005; Holtzheimer and Mayberg, 2011; Cif et al., 2013; Dougherty et al., 2016; Corripio et al., 2020). When viewing our results in this context, these results further support the hypothesis that low-frequency stimulation of cognitive networks involves a fundamentally different mechanism of action than that of DBS for movement disorders. While beyond the scope of this paper, such mechanisms might include changes to network oscillatory dynamics and/or synaptic plasticity.

In a study by Nayak and colleagues that used fUSI to study the effects of DBS in the thalamus of anesthetized rats, the authors reported increases to CBV lasting beyond the period of electrical stimulation (Nayak et al., 2021). The authors found that across a variety of pulse widths and voltages producing significant CBV responses, CBV remained elevated for approximately 2 additional minutes before returning to near-baseline levels. While this represents a relatively short time compared to the time period across which we found lasting effects, it should be noted that the authors used a much high stimulation current than our study and measured CBV in a 2D sagittal plane that covered nearly the entire brain. In contrast, we found that persisting effects of DBS were specific to brain regions with high anatomical connectivity to the MSN. It is therefore possible that by separating our signal by region, we were able to identify persisting effects that might have otherwise been obscured.

### Limitations and future directions

While the current study was performed in anesthetized animals, futures studies will investigate the effects of reduced NMDA function and MSN DBS in awake, behaving animals during memory-associated behavioral tasks (e.g., novel object recognition and Barnes Maze). The goal will be to determine if the observed ΔCBV within the septo-hippocampal network following theta- frequency MSN DBS is also associated with improved memory function, linking the present study with our previous study demonstrating improved memory following MSN theta-frequency DBS MK-801 treated animals (Zepeda et al., 2022).

Another limitation of the present study is that fUSI recordings were performed using a conventional 1-dimensional linear ultrasound transducer array that necessarily generates 2-dimensional pD vascular maps of the animal’s brain. As a result, other regions connected with the MSN besides the hippocampus and mPFC (e.g., amygdala, habenula, raphe nucleus) were not accessible from the selected sagittal 2-dimensional image plane. Recent studies are tackling this challenge using whole-brain 3-dimensional fUSI with either moving linear arrays (similar to the array used in our study), matrix arrays or raw column arrays (RCAs) (Rabut et al., 2019; Sauvage et al., 2020; Bertolo et al., 2021). Future studies can use these probes to cover volumes rather than slices of the mouse brain providing access to all areas of the septohippocampal network.

### Implications for neuromodulation

We observed that 7.7 Hz frequency stimulation of the MSN increased blood perfusion in the hippocampus both during and after stimulation. These effects were not observed with 100 Hz gamma-frequency stimulation and were still present even under conditions of pharmacologic NMDA antagonism. One important conclusion from the current study is that fUSI can capture small changes in brain hemodynamics and identify differences in the effects of DBS due to different stimulation frequencies. This strongly suggests the fUSI may be useful for future investigations using other neuromodulatory interventions as well, for example electroconvulsive therapy or transcranial magnetic stimulation.

## Data availability statement

The raw data supporting the conclusions of this article will be made available by the authors, without undue reservation.

## Ethics statement

The animal study was approved by University of Southern California, Institutional Animal Care and Use Committee (IACUC #21006). The study was conducted in accordance with the local legislation and institutional requirements.

## Author contributions

LC: Writing – review & editing, Writing – original draft, Visualization, Investigation, Formal analysis, Conceptualization. KA: Writing – review & editing, Writing – original draft, Visualization, Formal analysis, Data curation, Conceptualization. WC: Writing – review & editing, Writing – original draft, Investigation, Data curation. NZ: Writing – original draft, Writing – review & editing. EI: Writing – review & editing, Writing – original draft, Visualization, Validation, Software, Formal analysis. PP: Writing – original draft, Writing – review & editing, Visualization, Validation, Software. SS: Writing – original draft, Writing – review & editing, Supervision, Resources. CL: Writing – original draft, Writing – review & editing, Supervision, Conceptualization. VC: Writing – original draft, Writing – review & editing, Supervision, Resources, Methodology, Formal analysis, Data curation, Conceptualization. DL: Writing – review & editing, Writing – original draft, Supervision, Resources, Project administration, Methodology, Funding acquisition, Conceptualization.
